# State of the Art in the Antibacterial and Antiviral Applications of Carbon-Based Polymeric Nanocomposites

**DOI:** 10.3390/ijms221910511

**Published:** 2021-09-29

**Authors:** Ana M. Díez-Pascual

**Affiliations:** Universidad de Alcalá, Facultad de Ciencias, Departamento de Química Analítica, Química Física e Ingeniería Química, Ctra. Madrid-Barcelona, Km. 33.6, 28805 Alcalá de Henares, Madrid, Spain; am.diez@uah.es; Tel.: +34-918-856-430

**Keywords:** antibacterial activity, polymer nanocomposites, carbon nanomaterials, fullerenes, graphene, carbon nanotubes, synergic effects

## Abstract

The development of novel approaches to prevent bacterial infection is essential for enhancing everyday life. Carbon nanomaterials display exceptional optical, thermal, and mechanical properties combined with antibacterial ones, which make them suitable for diverse fields, including biomedical and food applications. Nonetheless, their practical applications as antimicrobial agents have not been fully explored yet, owing to their relatively poor dispersibility, expensiveness, and scalability changes. To solve these issues, they can be integrated within polymeric matrices, which also exhibit antimicrobial activity in some cases. This review describes the state of the art in the antibacterial applications of polymeric nanocomposites reinforced with 0D fullerenes, 1D carbon nanotubes (CNTs), and 2D graphene (G) and its derivatives such as graphene oxide (GO) and reduced graphene oxide (rGO). Given that a large number of such nanocomposites are available, only the most illustrative examples are described, and their mechanisms of antimicrobial activity are discussed. Finally, some applications of these antimicrobial polymeric nanocomposites are reviewed.

## 1. Introduction

Human beings are frequently infected by numerous microorganisms such as bacteria, fungi, viruses, algae, protozoa, and amoebas. Bacterial spread on materials has turned out to be a crucial matter in many fields, including therapeutic instruments, healthcare products, clinics, food storage, food packaging, dental equipment, and so forth [1]. To address this matter, different types of plastic materials are habitually sterilized. Nonetheless, microbes can contaminate these polymers when exposed to the atmosphere. While mortality from infections decreased considerably over the last decade owing to the commercialization of antibiotics, handling bacteriological contamination has lately become extremely difficult, given that many microbes have developed resiliency to antibiotics. For example, some of the Gram-positive (e.g., *Staphylococcus aureus*) and Gram-negative (*Escherichia coli, Pseudomonas aeruginosa*) bacteria are frequent medicine-resistant pathogens and constitute the main cause of hospital-acquired infections. A growing list of infections, i.e., pneumonia, tuberculosis, and gonorrhea, are becoming more difficult to treat, whereas antibiotics are getting less effective [2]. More than 25,000 patients from the EU die every year from hospital-acquired bacterial infections, mostly due to Gram-negative pathogens [3]. The costs caused by drug-resistant infections amount to more than 1.5 billion euros yearly, owing to the rise in health-related expenses. The circumstance is extremely severe, since antimicrobials have turned out to be a key tool for modern medicine and surgical operations. Therefore, there is an imperative necessity for developing novel antimicrobial materials that can inhibit bacterial proliferation [4].

One key mechanism for bacteria developing resistance is regular exposure to antibiotics. In this regard, nanomaterials with a size range of ≤100 nm in at least one dimension can encapsulate antibiotics. Nanomaterials can restore antibiotic efficacy because of their nanoscale functionalities. As carriers and delivery agents, they can reach target sites inside a bacterium by crossing the cell membrane, causing the leakage of the cellular components, and damaging its metabolism [5]. The smaller size of nanomaterials compared to their bulk counterparts modifies their physicochemical properties, leading to enhanced effectivity. The nanomaterial size can alter pharmacokinetics, specifically translocation, distribution, absorption, and elimination of the antibiotic [6]. The linkage of antibiotics with functional nanomaterials is critical, since their surface charge and their densities condition the effectiveness of bacterial killing. 

Recent work has investigated the use of different carbon nanomaterials as means of delivering antibiotics to hinder the resistant strain of *E. coli* [7]. Attachment of an antibiotic, tetracycline, to the nanomaterials was needed for the restriction of bacterial growth, demonstrating that the nanomaterials were carrying the antibiotic into the cells and destabilizing the efflux pump. The efficiency of carbon nanomaterials as antibiotic-delivery vehicles was found to be shape-dependent. Those with a needle-like shape showed better activity. Nanomaterials can successfully extend the life of current antibiotics, which makes them a key tool for supporting struggling antibiotic resistance through an efflux pump mechanism [7].

Some polymers have the capability to prevent the spread of microbes, which are named antimicrobial agents. They hinder cell development and induce cell apoptosis, and they can be classified into two groups according to their means of action [8]: (1) contact-active polymers, which use hydrophobic interactions, electrostatic forces, and the chelate effect; and (2) non-contact-active polymers, which release antibacterial agents, thus inducing cell death via linking or entering the cell wall. Polymers with intrinsic antimicrobial action include polypeptides such as poly-L-lysine (PLL) and poly-L-glutamic acid (PGA); polysaccharides such as cellulose (CL), chitosan (CS), and dextran (DEX); polyguanidines; and conductive polymers such as polypyrrole. Further, some polymers are modified in order to include antibacterial agents. Pendant groups frequently anchored to the polymer chains are NR_4_^+^ and OH^−^. The advantage of this approach is that the hydrophobicity, molecular mass, and charge may be tailored for specific applications. Additional information can be found in specific literature on this subject [9,10].

To date, many antimicrobial agents have been used as nanofillers in polymeric nanocomposites such as metallic and metal-oxide nanoparticles [11], as well as carbon nanostructures [12]. In particular, carbon nanostructures have gained a lot of interest owing to their exceptional properties and higher safety. Various mechanisms underlying the antibacterial activity of these nanomaterials have been reported [13], including physical/mechanical impairment, photocatalytic effect, oxidative stress, lipid extraction, isolation by wrapping, and the synergistic effect when they are combined with other antibacterial materials.

**Scheme 1:**
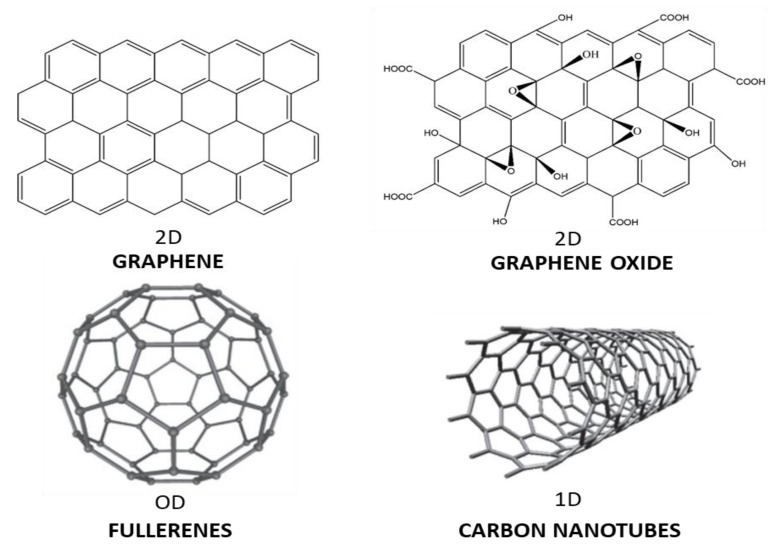
Chemical structure of carbon nanomaterials: 2D graphene and its derivative graphene oxide (GO), 0D fullerenes, and 1D carbon nanotubes (CNTs).

Carbon nanomaterials exhibit a large variety of shapes (Scheme 1), from 0D fullerenes to 1D carbon nanotubes (CNTs) and 2D materials, including graphene (G) and its derivatives graphene oxide (GO) and reduced graphene oxide (rGO). Further, new carbon-based nanomaterials are currently under investigation, such as mutated graphene-like nanomaterials, which have been found to be very effective for quick removal of organic pollutants from wastewater via surface adsorption and photocatalysis [14].

One characteristic of all carbon nanomaterials is the possibility of functionalizing them through non-covalent and covalent methods [15], which generally modifies their hydrophilic, electronic, optical, and mechanical properties. Non-covalent approaches are attained via π–π stacking, electrostatic forces, and Van der Waals forces. On the other hand, covalent functionalization can be performed via simple oxidation, leading to oxygen-containing groups, suitable to react with functional groups of other molecules or polymers. These procedures have already been extensively reviewed [16,17,18,19].

Fullerenes are symmetrical nanocarbon molecules with hollow structure (Scheme 1). They are caged compounds with fused-ring shape, composed of a varying number of polygons, and possess sp^2^ and sp^3^ hybridized carbon atoms. The most commonly known is C60, named as Buckminster fullerene [20]. It is made up of 20 hexagons and 12 pentagons, similar to a soccer ball. Due to delocalized π electrons, fullerenes have nonlinear optical responses, high electron affinity, and high transport charge ability. 

CNTs are rolled-up sheets of single-layer carbon atoms (Scheme 1). They can be single-walled (SWCNT) or multi-walled (MWCNT), and they display low density and very high electrical and thermal conductivity. However, they have a tendency to aggregate and form bundles; hence, functionalization with polymers [19] or other molecules is typically required for numerous applications.

G is a 2D flat atomic thick layer of sp^2^ carbon atoms with exceptional mechanical, thermal, optical, and electrical properties; molecular barrier ability; low density; little toxicity; and so forth [11]. However, the use of raw G has been restricted due to strong aggregation tendency, together with its hydrophobicity, which leads to insolubility in aqueous media. Thus, derivatives such as GO and rGO have been produced. GO is obtained via oxidation of G, and comprises COOH on the edges and epoxide, OH, and C=O moieties on the basal planes (Scheme 1). It has aqueous processability and amphiphilicity, and it can be partially reduced to G-like flakes, resulting in rGO [17,18]. Its electrical and thermal conductivity are lower than those of pristine G. Owing to the abovementioned motives, great effort has been dedicated to incorporating carbon nanomaterials into polymers to develop nanocomposites with improved properties due to synergistic effects.

Recently, some articles have discussed the antimicrobial activity of graphene and its derivatives [14,21,22,23]. In this review, the antimicrobial properties of nanocomposites containing polymers and carbon nanomaterials such as fullerenes, CNTs, G, GO, and rGO are reviewed. Owing to the vast amount of articles published on this topic, only the most illustrative examples are presented. In addition, applications of these nanocomposites are summarized.

## 2. Mechanisms of Antimicrobial Action of Carbon Nanomaterials

**Scheme 2:**
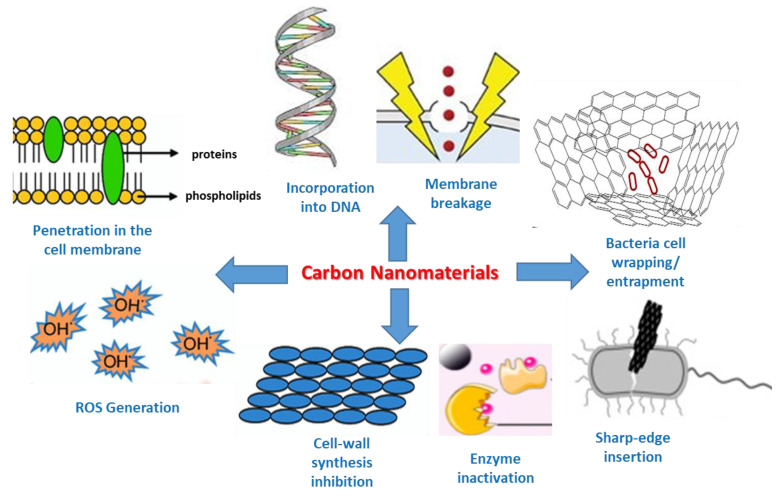
Illustration of the mechanisms responsible for the bactericide action of carbon nanomaterials.

Several mechanisms have been proposed to explain the antibacterial activity of carbon nanomaterials, as represented in Scheme 2. One of the most important chemical mechanisms is oxidative stress, which consists of the extra generation of reactive oxygen species (ROS). ROS are highly reactive oxygen-based molecules and free radicals such as peroxides (H_2_O_2_), superoxide (^•^O^−^_2_), singlet oxygen (^1^O_2_), hydroxyl radical (^•^OH), and so forth that cause enzyme inactivation and oxidative damage to cell components, including lipids, proteins, nucleic acids, etc. [24]. Additionally, electron transfer interaction can take place from bacterial membrane to carbon nanomaterials that easily accept electrons [25]. Several physical mechanisms have also been reported. An example is “sharp edge insertion”, in which nanomaterials such as G utilize their piercing boundaries to cut and penetrate the cell wall of the bacteria and provoke the outflow of intracellular material. Another is “direct contact” with the bacteria membrane, causing the membrane breakage [26]. Another potential mechanism is penetration of the cell membrane and lipid extraction. Thus, the nanomaterial sheets can enter the bacterial cell through the lipid bilayer. In addition, the nanomaterial can disrupt the protein–protein membrane connection and lead to malfunctioning [27]. Similar to membrane damage, disruption of DNA integrity can result in cell death. 

Bacteria can also be enclosed within the carbon nanomaterial and thus separated from their growth medium [28], leading to cell entrapment or wrapping, which inhibits cell development. Additionally, some carbon nanomaterials are inhibitors of the cell wall synthesis. For instance, some of them bind to the amino acids within the cell wall, preventing the addition of new units to the peptidoglycan layer.

### 2.1. Antimicrobial Activity of Fullerenes 

Fullerenes and their derivatives can inhibit bacterial growth and metabolism. Gram-positive bacteria are more susceptible to fullerenes, owing to their higher membrane permeation [29]. Another issue affecting the antimicrobial properties of fullerenes is electrostatic interaction, which changes with the fullerene form. Additionally, they can also generate ROS, including singlet oxygen, thereby destroying the bacterial cell, and they can avoid oxygen intake, depending on the concentration. Another mechanism for elucidating their antimicrobial characteristics is bacterial membrane damage, due to interaction with membrane proteins or other molecules. Thus, the outer membrane surface has a negatively charged lipopolysaccharide layer prone to interacting strongly with cationic fullerenes [30]. Consequently, positively charged fullerenes are very effective against Gram-negative bacteria such as *E. coli*. In addition, those with amino groups display stronger antimicrobial activity. They also seem to display environment-related selectivity. Thus, fullerenes have no toxicity or effects on anaerobic microorganisms, while having significant effects on microorganisms in soils containing a small amount of clay and organic matter [31].

### 2.2. Antimicrobial Activity of Carbon Nanotubes 

SWCNTs can display excellent antimicrobial activity, especially those with smaller size, hence larger specific surface area. The mechanism relies on their direct contact with bacteria membrane, thus altering its fluidity, causing oxidative stress, enzyme inhibition, and decreased transcription of various genes [32]. A large number of parameters including CNT size, agglomeration state, degree of functionalization, level of purification, concentration, period of contact, etc., significantly condition the antibacterial action of CNTs. The smaller the diameter, the better the penetration into the cell wall. The bacteriostatic characteristics of SWCNTs and MWCNTs have been compared, and it was found that SWCNTs show better performance, particularly those with surface functional groups (i.e., OH and COOH) [33]. The surface charge also has an impact on the biocide properties. Both positively and negatively charged tubes have an antibacterial effect due to ROS generation such as hydroxyl radicals. The CNT length also has a strong effect: in liquid media, the lengthier the SWCNTs, the more intense their outcome [34]. Another crucial parameter is their diameter: smaller ones can better interact with the bacteria wall, which is detrimental [35]. CNTs with small diameters connect to the microorganism at one termination, while those with big diameters (i.e., >15 nm) link to the microorganism by their sidewalls.

**Figure 1:**
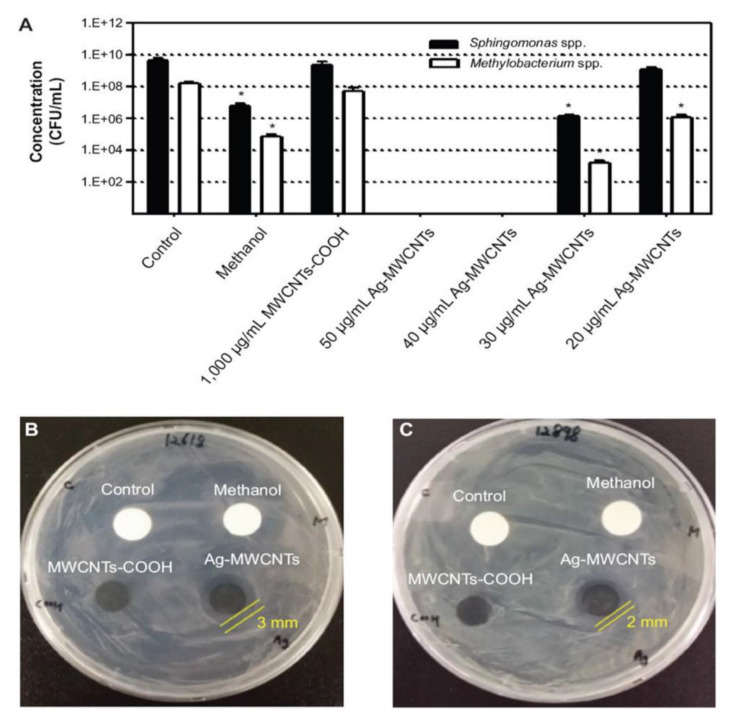
(**A**) Antibacterial activity of acid-treated multiwalled carbon nanotubes (MWCNTs-COOH) and different concentrations of Ag-MWCNTs against *Sphingomonas* spp. and *Methylobacterium* spp. * *p* < 0.05, one-tailed Mann–Whitney U-test. Data are representative of three experiments. (**B**) Evaluation of the inhibition zone of different samples against *Sphingomonas* spp. (**C**) Inhibition zone against *Methylobacterium* spp. Reproduced from Ref. [38].

Synergy is crucial for biocide action. For instance, CNTs and nanoparticles such as Ag, ZnO, and CuO have a great synergistic effect [36,37,38]. In particular, composites with MWCNTs and AgNPs have been produced and have showed excellent antimicrobial properties [38] against *Methylobacterium* spp. and *Sphingomonas* spp. (Figure 1), with high selectivity, since no cytotoxicity towards mammal cells was detected. 

### 2.3. Antimicrobial Action of Graphene, Graphene Oxide and Reduced Graphene Oxide

The antimicrobial action of graphene-related nanomaterials arises from membrane damage due to direct contact with its piercing boundaries and oxidation stress (ROS generation) by electron transfer [39]. The effects of G, GO, rGO, and graphite oxide on *E. coli* have been compared [40] under analogous conditions and followed the order: GO > rGO > G > graphite oxide. GO, with the smallest average size, had the highest defect density, and hence the toughest oxidative stress. Further, the amount of surface groups influences the biocide action [41]. GOs with lower level of oxidation generated more ROS by supporting the decomposition of H_2_O_2_ into ^•^OH.

Gram-negative bacteria such as *E. coli* are more resistant to membrane harm produced by rGO than Gram-positive ones such as *S. aureus,* since these do not have any outer membrane [28]. Size also influences the penetration of G into the lipid bilayer. Large-sized G adopted a perpendicular conformation to the cell wall, while small-sized ones took a parallel configuration [42]. Furthermore, the activity depends on the flake size and number of layers. Smaller flakes show greater cellular internalization and hence have more effect on the cell functionality. Additionally, G with a few layers has less antimicrobial activity than G monolayer [43]. Other features that condition the antibacterial activity are specific surface area and roughness. The bigger the surface area, the higher the number of locations accessible for bacterial contact. The rougher the surface, the higher the number of contacts with the bacteria. Additionally, the degree of dispersion of carbon nanomaterial in the medium has a great influence on the biocide effect [28]. Selective antibacterial effect of these nanomaterials towards human pathogenic bacteria has also been reported [44], which is a key point in order to avoid cell damage. 

## 3. Antibacterial Action of Polymer Nanocomposites with Carbon-Based Nanomaterials 

Polymeric nanocomposites contain a polymer or copolymer as matrix, having nanoparticles or nanofillers dispersed. Polymeric nanocomposites comprising carbon-based nanomaterials can be prepared by different methods such as melt compounding, solution blending, latex mixing, in situ polymerization, and electropolymerization [16]. In the following sections, a brief overview of different polymer/carbon-based nanocomposites with antibacterial activity will be provided. 

### 3.1. Antibacterial Properties of Nanocomposites Incorporating Fullerenes

**Scheme 3:**
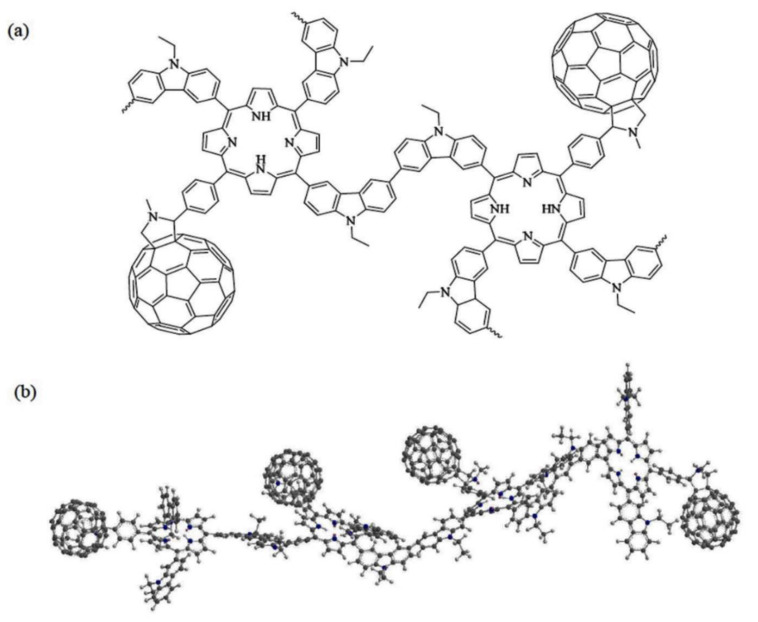
Schematic representation of porphyrin-C60 (**a**) and its polymerized structure (**b**). Taken from Ref. [46].

A few studies have investigated the antimicrobial properties of polymeric nanocomposites reinforced with fullerenes. For instance, Alekseeva et al. [45] found that polystyrene films filled with fullerenes had antibacterial characteristics against *S. aureus, E. coli*, and *C. albicans*. In fact, disk diffusion tests revealed zones without bacterial growth nearby the nanocomposite films, while the polymer alone did not show antimicrobial properties. The reason for microbial inactivation is believed to be the interaction of fullerene with the amino acids of proteins in the bacteria, causing cell membrane damage. Additionally, the bacterial inactivation persisted for one month. Other researchers investigated the effect of incorporating C60 or OH-C60 with polysaccharides such as CL, CS, and γ-cyclodextrin. It was found that γ-cyclodextrin/CS/fullerene nanocomposite had significant antimicrobic action versus vancomycin-resistant enterococci and was suitable for a diversity of uses, such as food wrapping. Porphyrin-fullerene C60 polymeric films (Scheme 3) also displayed antibacterial action versus S. aureus and *E. coli*. The number of S. aureus and E. coli bacteria was reduced by up to 4 logs after 30 and 60 min of irradiation, respectively [46]. The formation of ^•^O^−^_2_ was believed to be the main reason for the antimicrobial action. Consequently, porphyrin-fullerene films possess an active, elastic, photodynamic surface that can overwhelm microbes.

### 3.2. Antibacterial Properties of Nanocomposites with Carbon Nanotubes

**Figure 2:**
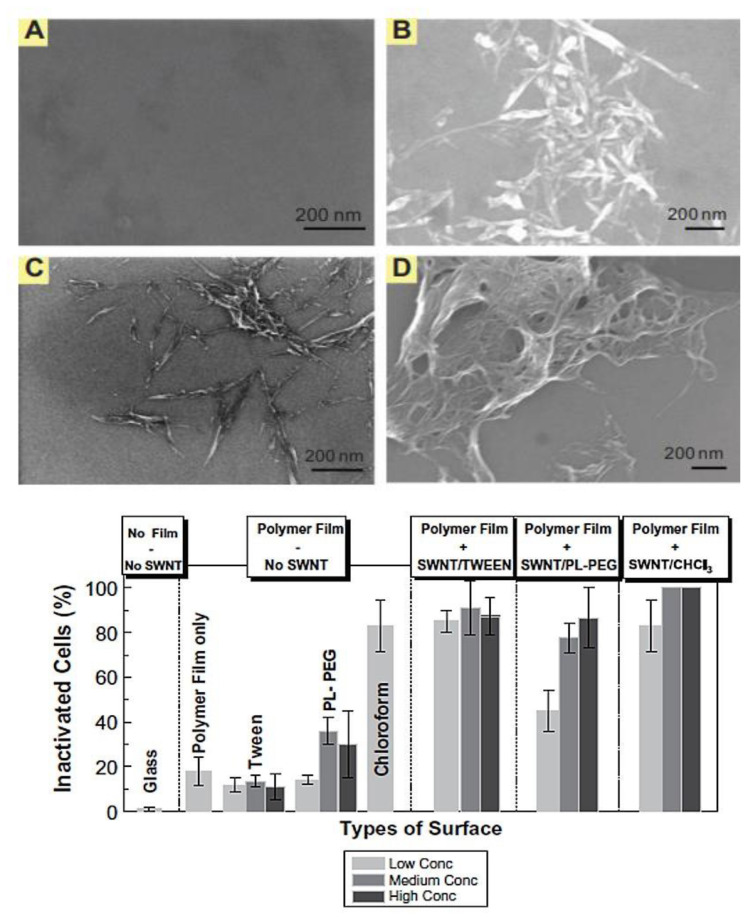
Top: SEM micrographs of (**A**) (PLL/PGA)_10_, (**B**) (PLL/PGA)_10_ + (PLL/SWCNT-Tween)_2_, (**C**) (PLL/PGA)_10_ + (PLL/SWNT-PL-PEG/PGA/PLL/SWNT-PL-PEG), and (**D**) SWCNT on (PLL/PGA)_10_. Bottom: Deactivation percentage of *E. coli* on various substrates. Controls are neat glass, a LbL film without SWCNT, a film with SWCNTs and Tween or PL-PEG, and a neat polymer film with SWCNTs. Taken from Ref. [47].

Nanocomposites filled with CNTs have been developed to modulate antimicrobial properties. It was found that the incorporation of AgNPs on MWCNTs into polyamidoamine caused bacteriostatic effects versus *E. coli*, *S. aureus,* and *P. aeruginosa*. Owing to the CNT-AgNPs’ interaction, the AgNPs were enclosed, and the Ag toxicity was reduced. Further, a synergistic behavior of both nanomaterials on the antibacterial activity was proposed. The antimicrobic action of SWCNTs layer-by-layer (LbL) assembled with polyelectrolytes such as PLL and PGA was assessed (Figure 2) [47].

SWCNT dispersion in water was attained using Tween 20, a nonionic biocompatible surfactant and phospholipid-poly(ethylene glycol) (PL-PEG), an amphiphilic biocompatible polymer. SEM micrographs of the LbL films showed that PLL-PGA without SWCNTs is smooth and featureless (Figure 2A), while single or little SWCNT bundles were found in SWCNT-Tween and SWNT-PL-PEG films (Figure 2B,C). When pure SWCNTs were deposited onto (PLL-PGA) from chloroform, a considerably higher agglomeration was found (Figure 2D). The deactivation rate of *E. coli* after 1 day of incubation on several substrates was analyzed (Figure 2). A very small amount of bacteria expired on neat glass. Films comprising raw polymers or those exposed to Tween and PL-PEG were almost innocuous, with deactivation rates lower than 20%. The coating with PL-PEG displays a certain toxicity (about 40% deactivation) at intermediate and elevated concentrations, whereas the film with SWCNT-Tween deactivates more than 80% of the bacteria for all the concentrations tested [47]. The film with SWCNT-PL-PEG also deactivates about 90% of bacteria at the maximum SWCNT loading, though is not as effective. A biocide activity of 90% against *S. epidermidis* was also attained, which demonstrates the suitability of SWCNT/PLL/PGA thin films as antimicrobial biomaterials. Additionally, SWCNTs bonded to PLL via covalent bonding had biocide action versus *E. coli, S. aureus*, and *P. aeruginosa*. 

In CS/CNT nanocomposites, antibacterial properties rise with increasing CN loading; these nanocomposites exhibit a clear porous structure with elevated water absorption [48], and the inhibition zone diameters are close to those recorded for common antibiotics. On the other hand, gelatin/MWCNT nanocomposites also display antibacterial effect against Gram-negative and Gram-positive bacteria. The use of gelatin in nanocomposites is limited owing to its poor mechanical properties, which may be solved through the incorporation of CNTs [49]. Polylactic acid (PLA)/ CNT/AgNP nanocomposites have also been developed, which have showed antibacterial properties against *S. haemolyticus* due to synergistic effects [50]. 

The application of conducting polymers as antibacterial agents is also a very promising strategy for the development of novel antibacterial systems [51]. The electrostatic interactions between the conducting polymer and the bacteria lead to microorganism adhesion to the polymer surface. Subsequently, diffusion of nanoparticles and active counter-ions particles towards the cytoplasmic membrane takes place, followed by their permeation into the cell, which ultimately results in cell death. In particular, polypyrrole (PPy) has been regarded as an effective antimicrobial polymer. However, the aggregation of polymer chains is a critical drawback that needs to be addressed. A plausible solution is combination with nanomaterials such as CNTs. Thus, PPy/CNT nanocomposites show a good combination of conductivity, antibacterial activity, and strong absorption of light in the NIR region. Tondro et al. [52] developed this type of nanocomposites and applied them in a phototherapy treatment based on IR light irradiation. The straight incidence of irradiation boosts the physical rupture of the cells by the CNTs, the electrostatic attraction of PPy to the bacterial cells, and the extra generation of ROS. Therefore, a reduction in cell viability is observed, as a consequence of protein and DNA leakage and ROS production that inhibit essential processes in the bacteria. The development of alternative antibacterial agents based on conducting polymers and CNTs represents a promising strategy to circumvent the increasing resistance to antibiotics. This can be further improved by physical methods such as phototherapy and electrical excitation to avoid restrictions related to the anchoring of microorganisms to the polymer surface, which is of great interest in different areas such as medical, food, and textile industries. 

**Figure 3:**
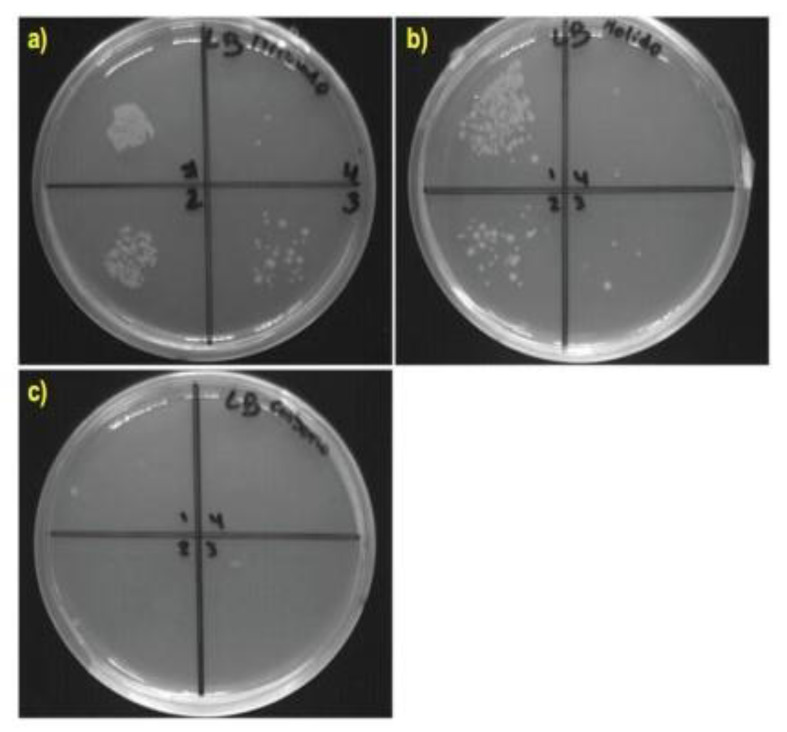
Images showing the colonies of *E. coli* bacteria left on the surfaces of neat LDPE (**a**), ball-milled LDPE (**b**), and LDPE/MWCNT nanocomposite (**c**). Taken from [53].

**Figure 4:**
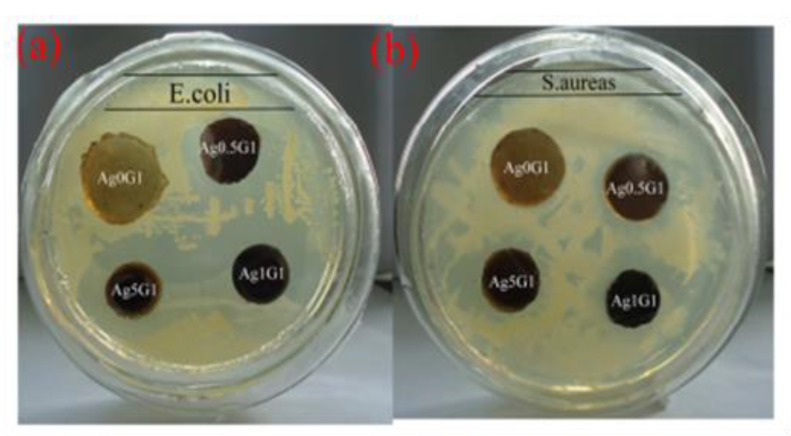
Images demonstrating the antibacterial activity of PNiPAM hydrogels against *E. coli* (**a**) and *S. aureus* (**b**). The nanocomposites with different Ag:G mass ratios (0.5:1, 1:1, and 5:1) are named as Ag0.5G1, Ag1G1, and Ag5G1, respectively. Taken from Ref. [58].

Benigno et al. [53] prepared low-density polyethylene (LDPE)/MWCNT nanocomposites, which showed biocide action versus *E. coli*. In particular, the nanocomposite with 1 wt% MWCNTs had strong antimicrobial activity (Figure 3). It seems that the substrate influences bacteria adhesion and also changes their metabolism. Thus, worse adhesion is found for the nanocomposite, and hence no *E. coli* bacteria remained (Figure 3c), while 2.6 × 10^7^ CFU/mL were found for neat LDPE (Figure 3a) and 4 × 10^6^ CFU/mL for a ball-milled LDPE that was used as control (Figure 3b). Aslan et al. [54] developed poly(lactic-co-glycolic acid) (PLGA) nanocomposites with SWCNT concentration up to 2 wt%, which effectively reduced the viability of both *E. coli* and *S. epidermidis* (up to 98% at 2 wt% loading). Shorter SWCNTs were more toxic, probably owing to their higher number of exposed tube terminations. This study demonstrates the potential of PLGA/SWCNT as an antimicrobial biomaterial.

### 3.3. Antibacterial Properties of Nanocomposites with Graphene and Its Derivatives

A number of studies have investigated the effect of nanocomposites based on polymeric matrices and graphene or its derivatives. For instance, polymethyl methacrylate (PMMA) matrix filled with 1 and 2 wt% Ag nanoparticles and GO displayed strong antimicrobial action versus *S. aureus, E. coli*, and *S. mutans* [55]. The nanocomposite comprising GO and Ag wraps the bacteria via a direct contact, assisted through H-bonding formation with the proteins of the membrane, hindering them and producing programmed cell death. There is a synergy of GO and Ag nanomaterials in enhancing biocide effect, as found for numerous G-based hybrid nanocomposites [56].

Furthermore, fibers of PMMA comprising GO have been manufactured, which showed antibacterial action versus *E. coli* ascribed to ROS generation. The maximum bactericide action (about 85%) was found for the nanocomposite with 8 wt% GO content [57]. PNiPAM nanocomposite hydrogels based on Ag/G mixtures (0.5:1, 1:1, and 5:1 *w*/*w*) with acrylic acid crosslinked with N,N′-methylene bisacrylamide have also been synthesized [58]. The nanocomposite with Ag:G mass ratio of 5:1 displayed optimum performance, with excellent biocompatibility, high swelling ratio, and superior antimicrobial activity (Figure 4). It was reported that the greater the Ag concentration, the stronger the biocide action. Moreover, Ag/G nanocomposites showed considerably stronger antibacterial properties than pristine G or Ag nanomaterials, since G can avoid nanoparticle agglomeration. Similar PNIPAM hydrogels filled with GO or GO/CNT nanocomposites were synthesized by free radical polymerization [59], and the results corroborated the outstanding antimicrobial activity against *P. aeruginosa*. 

Antimicrobial nanocomposites comprising biocompatible synthetic polymers have been prepared. For instance, solution-casted poly(vinyl alcohol) (PVA)/GO nanocomposites with 1, 5, and 10 wt% loading had good antimicrobial properties against *E. coli* and *S. aureus* [60]. Additionally, stronger action was found against the Gram-positive bacteria, as previously reported by other authors, since the structure of their membrane is different. Thus, Gram-negative bacteria has an additional peptidoglycan layer in the membrane, which offers an additional barrier for microbial penetration [61]. PVA/Ag/GO nanocomposites synthesized via chemical reduction combined with solution casting had strong antibacterial action versus *E. coli* and *S. aureus* owing to synergy of both nanofillers [62]. Additionally, the antibacterial performance was influenced by the GO/Ag content and the period of time [63]. Samples with GO/Ag content lower than 1 wt% did not affect the growth of *S. aureus* and hardly affected *E. coli*, while those with 2 wt% led to a strong reduction in bacterial growth, and nanocomposites with 5 wt% GO/Ag inhibited completely both bacteria after 24 h. Usman et al. [64] added starch as a reducing agent, thereby forming in situ rGO, intercalated within PVA. In this case, the antibacterial outcome arose from the synergy of rGO and Ag dispersed into the polymer matrix comprising starch.

Antibacterial poly(N-vinylcarbazole) (PVK)-based nanocomposites with GO have also been developed via bulk polymerization, and their effects were assessed versus *C. metallidurans* and *E. coli* (Gram-negative) as well as *R. opacus* and *B. subtilis* (Gram-positive) [65]. The PVK–GO nanocomposites had stronger antimicrobial properties than pristine GO. The mechanism of inactivation was related to the wrapping of the bacteria by the nanocomposite, which lessened the metabolic activity of the bacteria and lastly caused cell decease. *S. aureus* was less resistant to these nanocomposites than *E. coli*.

Polylactide acid (PLA) has also been combined with GO and its mixtures with nanoparticles, resulting in nanocomposites with improved antibacterial action versus *S. aureus* and *E. coli* [66,67,68,69]. Several parameters including nanocomposite preparation technique, nanofiller content, morphology, and level of nanofiller dispersion within the matrix condition the final properties. In general, the efficiency grows with increasing GO concentration and can also be enhanced via application of an external electrical stimulus. Ternary PLA/GO/ZnO nanocomposites prepared by solution-blending technique showed durable UV resistance and biocide action with small GO/ZnO contents [67]. PLA/GO/Ag hybrids were prepared via in-situ polymerization followed by mechanical blending, and the effects of the GO/Ag content were examined [69]. The antibacterial efficiency increased up to 99% as the loading augmented up to 2.0 wt%. GO was believed to improve the antibacterial action of Ag nanoparticles in the matrix owing to improved distribution of Ag onto GO surfaces and hence stronger Ag-cell wall interactions.

**Figure 5:**
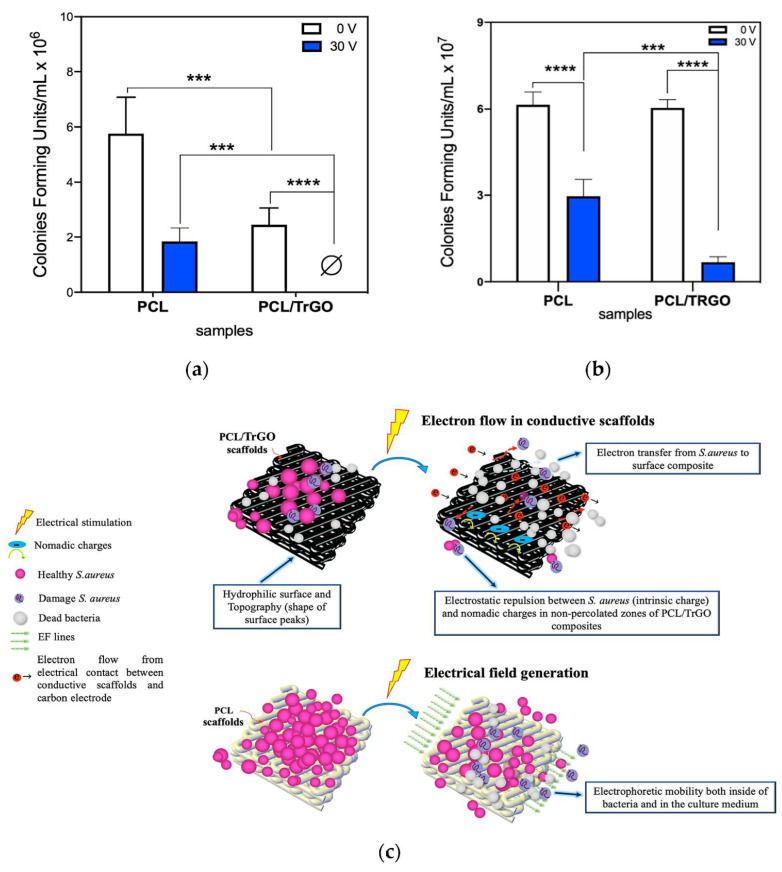
(**a**) Colony-forming units for the experiments performed at 0 V and under electrical stimuli (30 V) for neat PCL and PCL/rGO nanocomposite. (**b**) CFU in scaffolds taken from the bioreactor after 3 h of incubation at 0 V and 30 V. *** and **** denote a significant difference with a value *p* < 0.001 and *p* < 0.0001, respectively, with the control sample PCL 0 V and between the samples indicated, n = 3 independent experiments were performed in triplicate per sample (**c**) Depiction of the probable mechanism of antibacterial action for PCL/rGO without electrical field and for PCL and PCL/TrGO under stimulation at 30 V. Taken from Ref. [71].

Another biodegradable polymer, polycaprolactone (PCL), shows antibacterial activity after mixing with rGO/Ag [70]. PLC/rGO/Ag nanocomposites with a mass ratio of 94:5:1 were synthesized by solution casting and had stronger biocide action than binary PLA/Ag or PLA/rGO, ascribed to a direct contact mechanism by mechanical breakage of the cell membrane in the presence of Ag well-dispersed onto the rGO surface. PCL scaffolds with conductive rGO nanoparticles have been prepared via a 3D-printing process [71]. A noteworthy antibacterial effect was found upon application of DC to the percolated scaffolds: under electrical stimulation, a 45% decrease in *S. aureus* colonies was attained in neat PCL, which is electrically nonconductive, compared to PCL without electrical stimuli (Figure 5a). This behavior was attributed to electrophoretic effects, given that the unique membrane of *S. aureus* is subtle to exterior electrical fields, which cause damage and release of intracellular content, reducing cell viability. In the PCL/rGO scaffolds, 100% bacterial colonies were eradicated under electrical stimulation. The surface topography is another key parameter governing bacterial adhesion. Thus, a certain roughness was found in PCL/rGO scaffolds surfaces that contributed to the detachment of bacteria in the lack of electrical stimulation. A potential antibacterial mechanism is related to the flow of electrons between the conductive scaffolds and the carbon electrode, since the electrophoretic forces within the nanomaterial produced an antiadhesive effect, which increased bacterial mobility. This effect was corroborated via counting the CFU from the reactor solution, as displayed in Figure 5b. Under electrical stimuli, a noteworthy reduction in CFU was found, equal to about 49% and 89% for neat PCL and PCL/rGO, respectively, compared with the control. The hypothetical antibacterial mechanisms are depicted in Figure 5c.

PVDF/Ag/GO fiber mats with a GO content of 1 wt% and Ag nanoparticle contents of 0.5, 1, and 2 wt% have been synthesized via electrospinning [72]. Pure PVDF showed no antibacterial activity, since its hydrophobicity helps the bacteria to adhere onto its surface. However, PVDF/Ag nanocomposites can hinder bacteria from linking to the membrane surfaces via release of Ag^+^ ions that interact with the SH moieties of DNA enzymes. The addition of 1 wt% GO further improved the bactericidal activity against *E. coli*, particularly at 2 wt% Ag loading, attributed to a better Ag dispersion within GO, and the cutting edge mechanism. As mentioned earlier, GO can pierce the bacterial membrane and provoke the outflow of intracellular constituents. Besides, a synergy of Ag and GO nanofillers should take place [73]. 

Nanocomposites of polyethylene glycol (PEG)-functionalized GO with Ag nanoparticles were synthesized via a rapid and environmentally friendly microwave irradiation method [74], at different radiation periods and with different Ag nanoparticle sizes (8 and 50 nm). They showed outstanding microbial activity against *E. coli*, and those with the smaller nanoparticles showed better antibacterial activity. Smaller nanoparticles adsorb more easily onto the surface of the cell, which can impact the membrane permeability and origin its rupture. Besides, bacteria could be enveloped by the ternary, which would limit bacterial nutritional supplementation, and consequently, their metabolism. They can also weaken the *E. coli* cell wall, and provoke the rupture of the cell membrane via a synergetic effect of both nanofillers. 

**Figure 6:**
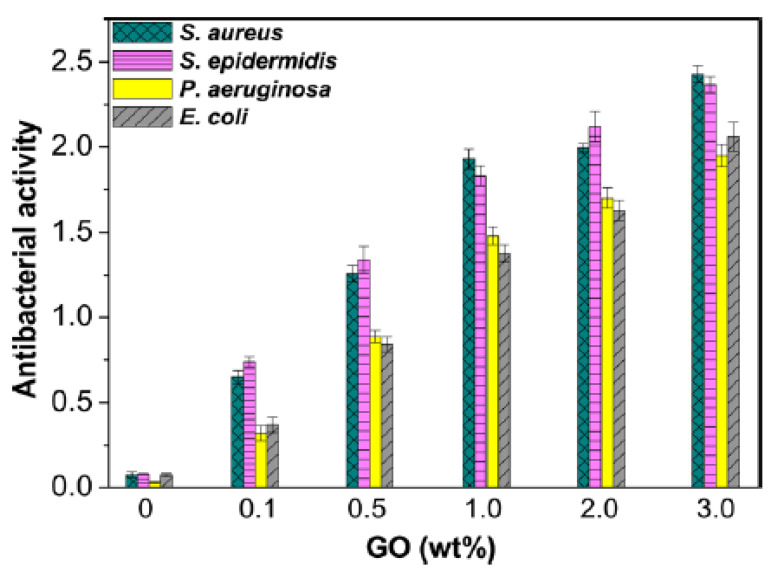
Antibacterial action of PPF/PEG-GO nanocomposites with different GO loadings against *S. aureus, S. epidermidis, P. aeruginosa*, and *E. coli*. Taken from Ref. [13].

Poly(propylene fumarate) (PPF), a biocompatible and biodegradable copolyester, has been combined with GO previously functionalized with PEG via a noncovalent strategy [13]. The antibacterial performance versus *P. aeruginosa, E. coli, S. aureus*, and *S. epidermidis* increased intensely with growing GO content (Figure 6), and the nanocomposite with 3.0 wt % GO loading showed the highest antibacterial activity.

The antibacterial effect was more intense against Gram-positive bacteria. Thus, in this type of bacteria, at 1.0 wt% GO loading, the logarithmic ratio of viable cell count in the control to that in the nanocomposite was equal to or higher than 2, while for the Gram-negative bacteria, this value was overpassed at 3 wt% GO content. Hardly any difference between the effect on *S. aureus* and *S. epidermidis* was detected, and the same behavior was found when comparing *E. coli* and *P. aeruginosa*. The same mechanism was proposed to explain the antibacterial activity of poly(glycolic acid-co-propylene fumarate) (PGA-co-PPF)/GO/hydroxyapatite nanorods (HA) nanofibers prepared via electrospinning [75]. (PGA-co-PPF) did not show antibacterial action on the bacteria, though nanocomposites comprising GO showed activity towards both types, which was more intense versus *S. aureus*. Conversely, PGA-co-PPF/HA nanocomposites did not affect *S. aureus* and only had a small impact on *E. coli*. It seems that the blend of HA and GO increases the antimicrobial effect.

Some studies focused on nanocomposites based on polysaccharides such as CL, CS, alginate, and starch, filled with G or its derivatives, have been reported [76,77,78,79,80,81,82,83,84,85,86]. Membranes of CL, a linear homopolymer of *β-D* glucose with GO and Ag nanoparticles, were synthesized by a process comprising two stages [76]. First, the mixture was coagulated to fabricate membranes comprising different GO loadings. Then, Tollens’ method was applied to synthesize in situ the AgNPs [87]. The presence of GO enhanced the deposition of Ag owing to the electrostatic interactions with surface GO groups. These nanomembranes were very effective towards *S. aureus* and *E. coli*.

Agar, a mixture of agarose and agaropectin, has been reinforced with ZnO, Ag-NPs, and rGO nanosheets [77]. Chemical interaction/complexation occurred between functional moieties of rGO and the hydroxyl groups of the inorganic nanoparticles. This nanocomposite revealed superior antibacterial effect (close to 95%) against *S. aureus* and *P. aeruginosa*, for a long period of about 90 days, ascribed to a synergy of the three nanofillers. Nanocomposite films based on furcellaran, a type of high-molecular-weight polysaccharide with sulphate groups, GO, carbon quantum dots (CQDs), and maghemite nanoparticles, were developed via solution casting [78]. The nanocomposites demonstrated a noteworthy inhibition on the proliferation of *S. aureus* and *E. coli*. 

**Figure 7:**
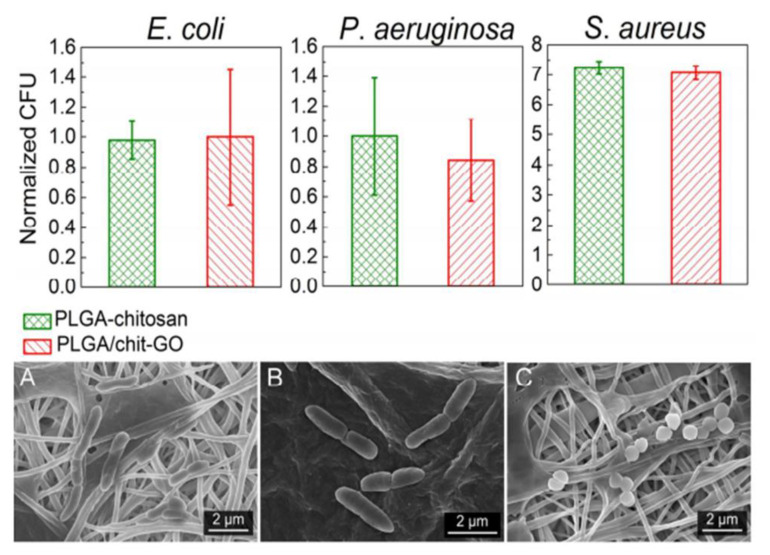
Top: CFU on PLGA-CS (control) and PLGA/CS-GO after exposure to *E. coli, P. aeruginosa,* and *S. aureus*. Bottom: SEM micrographs of *E. coli* (**A**), *P. aeruginos*a (**B**), and *S. aureus* (**C**) cells that have been exposed to the PLGA/CS-GO fiber mats. Taken from [79].

CS presents antibacterial and antifungal properties, which depend on molecular weight, degree of acetylation, and pH: high molecular weight and low degree of acetylated CS is less effective against Gram-negative bacteria [88]. Its bactericide action is ascribed to the interaction between its positively charged NH_3_^+^ groups and negatively charged bacteria membranes. This electrostatic interaction changes the membrane permeability, causing osmotic disproportions, and hence hinders the bacteria growth. It also causes the hydrolysis of the peptidoglycans in the bacteria wall, causing the release of the cell components. A mixture of poly(lactide-co-glycolide) (PLGA) and CS electrospun fiber mats have been modified with GO–AgNPs through reaction between the NH_2_ moieties of the PLGA–CS fibers and the COOH moieties of GO; a carbodiimide hydrochloride derivative and N-hydroxysuccinimide were used as cross-linkers. [79]. The ternary nanocomposites successfully deactivated *S. aureus*, *E. coli*, and *P. aeruginosa*, showing better activity than PLGA-CS mixtures (Figure 7). The PLGA-CS/GO-Ag inhibits almost completely *E. coli* and *P. aeruginosa*, but has weaker effect (close to 80%) against *S. aureus*. This is ascribed to the different assemblies of the bacterial wall. SEM micrographs confirm that Gram-negative bacteria stuck on PLGA-CS/GO-Ag fibers collapse and lose their physical integrity (Figure 7A,B). The characteristic structure of the Gram-negative bacteria, with a rod-like shape, is not found, and just a small amount of cell debris can be observed. Conversely, very little debris can be observed in the micrographs of *S. aureus* (Figure 7C). Bactericidal effect through a direct contact mechanism was proposed.

CS/rGO composites have been prepared by an environmentally friendly procedure. The rGO was synthesized by reduction with caffeic acid and afterwards distributed into CS. The films with rGO content in the range of 20–30 wt% of rGO displayed a rise of inhibition between 54% and 82% [80], ascribed to their ability to disrupt the cell membrane and provoke oxidative stress. CS/GO/TiO_2_ nanocomposites were also manufactured and exhibited almost complete inhibition versus *A. niger* and *B. subtilis* [81] when their ratio was 20:1:4. The activity was related to cell wall harm induced by the nanocomposite. CS/polyvinylpyrrolidone (PVP)/GO were also prepared, and both covalent interactions via nucleophilic substitution reaction between the epoxy moieties of GO and amino groups of CS and non-covalent interactions by H bonding between the OH groups of GO and the NH_2_ moieties of CS were analyzed [82]. When the GO content was low, the degree of crosslinking with CS was low, and it remained as an individual nanofiller. Conversely, when the GO content was high, the crosslinking level was high, and it formed aggregates that worsened the nanocomposite’s physical characteristics. Better bactericidal capability versus *S. aureus* and *E. coli* was observed for the nanocomposite with 25 vol% PVP and 1 wt% GO. Although the mechanism behind the antimicrobial effect of CS is still not entirely understood, it could be ascribed to its aptitude for passing the bacteria cell wall through permeation and development of a polymer coating on the surface of the cell wall, therefore avoiding nutrients entering the cell [83]. In addition, its NH_3_^+^ are prone to interact with the negative charges of the cell membranes, causing the release of proteins and other constituents from the interior of the cell. Moreover, this polysaccharide can modify the phospholipid bilayer structure in the cell membrane, altering its degree of permeation, henceforth causing the liberation of cellular components.

Starch is a blend comprising 20–30% of linear amylose with α-(1,4)-linkages and 70–80% highly branched amylopectin, with α-(1,4) and α-(1,6) linkages. Starch-reduced GO (SRGO) linked to polyiodide nanocomposites have been prepared via a hydrothermal treatment [84]. The nanocomposites exhibited good antibacterial activity versus *E. coli* and *S. Aureus*, while neat SRGO did not display any inhibition. The mechanism proposed was the continued release of polyiodide, which disrupts the bacterial membrane. 

Alginates are ionic-block copolymers comprising sections of consecutive *β-D*-mannuronic acid monomers (M-blocks), sections of *α-L*-guluronic acid (G blocks), and regions of scattered M and G units. Alginates crosslinked with Ca^2+^ cations and GO were prepared by mixing ZnCl_2_ with a GO/Ag dispersion in water under agitation and then solution casting [85]. The addition of 1 wt% GO produced a significant biocide effect versus *S. aureus* and *S. epidermidis*, while it was not toxic for human cells. An analogous method was used to synthetize alginates crosslinked with Na^+^ that incorporated GO, which showed higher antibacterial action versus the aforementioned bacteria for identical GO concentration [86].

Polyhydroxyalkanoates (PHAs) are a family of bacteria-based biodegradable plastics produced from natural resources such as cane sugar. Their synthesis typically occurs during fermentation under nutrient-limiting conditions. The most commonly investigated is poly(3- hydroxybutyrate) (PHB), a polyester with properties comparable to synthetic polypropylene [89,90]. Further, its copolymerization with 3-hydroxypentanoic acid produces poly(3-hydroxybutyrate-*co*-3-hydroxyvalerate), abbreviated as PHBV, another biocompatible green thermoplastic. PHBV/GO films with 1 wt% loading have been manufactured via solvent casting with the purpose of improving thermal and antibacterial properties as well as film wettability. The results showed that PHBV/GO exhibited better biocide action versus *S. aureus* than PHBV/carbon nanofibers (CNFs) [91]. Following the same approach, ternary PHBV/cellulose nanocrystals (CNC)/GO nanocomposites with CNC/GO weight percentages of 1:0.5 and 1:1 were prepared [92], and their properties compared with binary PHBV/GO and PHBV/CNC nanocomposites. The antibacterial activity for the binary nanocomposite with 0.5 wt% GO was higher than 99% versus *E. coli* and *S. aureus*. Superior performance was found for a PHBV/CNC/GO (98:1:1 wt%) nanocomposite that completely eradicated the growth of both bacteria, ascribed to cell membrane damage by oxidative stress or free radicals and the synergy of CNC covalently linked to GO by physical mixing. PHBV/rGO/ZnO hybrids with 3, 6, and 9 wt% nanofiller concentration were prepared by melt blending [93]. Prior to the hybrid synthesis, the concurrent reduction of Zn(O_2_CCH_3_)_2_ and GO at 20:1 ratio was performed, leading to the ZnO-GO nanocomposite. A noteworthy antimicrobial effect versus *E. coli* was observed, attributed to the intimate contact between the hybrid nanocomposite and the bacteria surface.

A comparison between the antimicrobial activity of some common antibiotics, nanoparticles, and polymeric nanocomposites with carbon-based nanomaterials is summarized in Table 1. 

## 4. Applications for Antimicrobial Polymeric Nanocomposites with Carbon Materials

Polymeric nanocomposites with carbon nanomaterials have great potential for numerous uses. For instance, they are widely examined for food packaging due to their extraordinary antimicrobial effects against most types of microbes [103]. The exceptional mechanical properties of carbon nanomaterials make them very suitable for development of active/smart packaging materials, since their incorporation in films can lessen the proliferation of microorganisms by eliminating or dropping oxygen in the interior of the wrapping, thus expanding the product shelf life and simultaneously reducing weight. High-fat foods can be wrapped by fullerene derivatives that have antioxidant properties [104]. One of the key uses of CNTs in the field of smart packaging is connected to the arena of sensors. CNTs can be used in sensors that monitor the state of the food, so that when an alteration occurs, it is reflected in a change in the packaging color, cautioning consumers about the lifetime of the product [105]. Likewise, CNTs can instantaneously detect some microbes that spoil foodstuffs.

Numerous cases of smart packaging comprising biopolymers and graphene nanostructures have been recently described [106]. The incorporation of G usually enhances the mechanical, barrier, thermal, and antimicrobial properties of the resulting nanocomposites. For example, CS/GO nanocomposites display optimal mechanical and barrier properties, and they restricted the proliferation of both *E. coli* and *B. subtilis* [107].

GO and clove essential oil have been incorporated into PLA by solvent casting [108]. The inclusion of GO enhanced the elasticity of the nanocomposite films by dropping the number of pores, thereby decreasing the permeability to oxygen, and the glass transition temperature. The nanocomposites displayed exceptional effectivity versus *E. coli* and *S. aureus*. All the mentioned features indicate the extraordinary capacity of GO and G for use in food storing. Fe_2_O_3_ coated GO/CS hydrogels were fabricated by co-precipitation followed by gel casting method [109]. The nanocomposites showed better thermostability, mechanical strength, and antimicrobial action versus *S. aureus, E. coli*, and *C. albican* and were found to be suitable for application in the food industry.

Nanocomposites with carbon nanomaterials also have many uses in the medical area [49,54], including wound-healing materials, drug delivery, and tissue engineering. In particular, nanocomposites with G may have applications in speeding up wound healing, lessening contaminations, and favoring proper curing without graze development. For instance, acrylic acid hydrogels crosslinked with N,N′-methylene bisacrylamide incorporating different Ag/G mass ratios have been developed. The hydrogel with an Ag:G ratio of 5:1 was biocompatible and exhibited elevated swelling ratio and superior biocide combined with exceptional mechanical performance, resulting in quicker wound-healing process [58]. 

**Figure 8:**
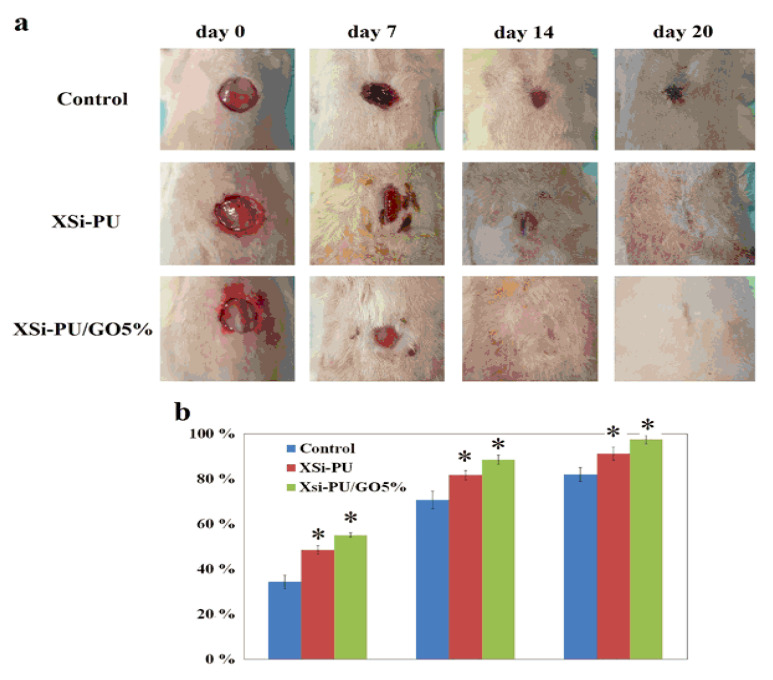
Pictures (**a**) and closure percentage (**b**) of wounds treated with gauze (control), polyurethane/siloxane membrane (XSi-PU), and the nanocomposite with 5 wt% GO (XSi-PU/GO5%) during the wound healing for 20 days. According to the analysis of variances, * *p* <  0.05, values are significantly different from the previously compared group. Taken from [110].

Similarly, antibacterial polyurethane (PU)/siloxane membranes incorporating GO nanoplatelets were prepared by the sol–gel technique, and displayed optimal biocompatibility with fibroblasts and better wound closure capacity [110]. The crosslinked siloxane areas and the GO nanoplatelets embedded within the PU chains provided the mechanical strength required for dressings. Additionally, the blending of hydrophilic and hydrophobic parts in the dressing allowed appropriate wound exudation management (Figure 8). 

In fact, better performance was observed for the membrane containing 5 wt% GO (XSi-PU/GO5%) than for the dressing without GO (XSi-PU) for the same period of time. For instance, on the 7th day, the wound enclosed by XSi-PU/GO5% membrane displayed about 55% wound healing, while control groups and XSi-PU treated wounds displayed values of 34% and 48%, respectively. On the 20th day, the wounds covered with the indicated nanocomposite displayed almost complete wound healing, whereas XSi-PU membrane and cotton gauze led to 91% and 81% decreases in wound size, respectively. SWCNTs and MWCNTs complexed with chitosan also improved the re-epithelialization of wounds; the most effective in the healing treatment was the nanocomposite with 1 wt% MWCNTs, due to the increase in the amount of deposited collagen in the wound [111]. On the other hand, antibacterial PU electrospun nanofibers with CNTs developed via an environmentally friendly approach using ethanol as the dispersion solvent exhibited excellent hemocompatibility and inferior hemolysis percentages, and they were found to be suitable as wound dressing materials [112]. 

Additionally, nanocomposites comprising G and polymers have been used for drug delivery. For instance, a mixture of polysebacic anhydride (PSA) and GO was developed by Gao et al. [113], and its effectiveness for the liberation of levofloxacin, a bactericidal drug, was examined. The PSA/GO nanocomposites required much more time for drug liberation than the raw polymer, as well as exceptional antibacterial efficacy without any toxic outcome. In addition, a PCL/G fiber comprising chlorhexidine (CHX), an antibacterial agent, was manufactured through melt rotation. The hybrid fibers showed excellent biocide performance and were crucial to minimize the risk of infections after operation [114]. 

In another study, molecularly imprinted polymers (MIPs) were manufactured via in situ polymerization in the presence of CNTs and were found to be appropriate for the electroresponsive liberation of a drug, diclofenac sodium salt [115]. In vitro tests, performed in water solution, revealed the optimal aptitude of the MIPs filled with CNTs to release the drug continuously, in a better way than the nonimprinted materials. Other researchers developed electroresponsive gelatin/MWCNT microgels with the aim to liberate the same drug upon application of an external voltage [116]. Several MWCNTs’ contents (up to 35 wt%) were used to select the CNT amount that offered the best sensitivity. At pH 7.4, the external electric field produced a decrease in the swelling rate from 1350% to 420%. Drug liberation tests revealed the potential of thermoresponsive nanocomposites to tailor the drug release over time. 

Owing to their mechanical stability, nanocomposites with carbon nanomaterials and polymers have been used in numerous tissue engineering studies. For example, a 3D CS/GO scaffold has been designed for bone tissue engineering [117]. The incorporation of GO enhanced pore development, mechanical properties, and bioactivity, thus promoting the possibilities for in vitro and in vivo applications. Several composite scaffolds including hydroxyapatite (HA) and collagen have been prepared via an in situ precipitation technique. The resulting scaffolds exhibited better strength and porosity than pure collagen, and thus, it is a hopeful approach for bone tissue engineering. Further mechanical improvement can be attained via the addition of CNTs. In this regard, collagen has been covalently bonded onto CNTs via creation of amide bonds. Afterwards, a HA coating was deposited onto the collagen-g-CNTs. Due to the chemical bonding, the flexural strength and fracture toughness of the collagen-g-CNT/HA nanocomposite with 3 wt% CNT increased by about 74% and 275%, respectively, compared to those of neat HA [118]. In addition, improved cell adhesion and growth were detected for the ternary nanocomposites. 

**Scheme 4:**
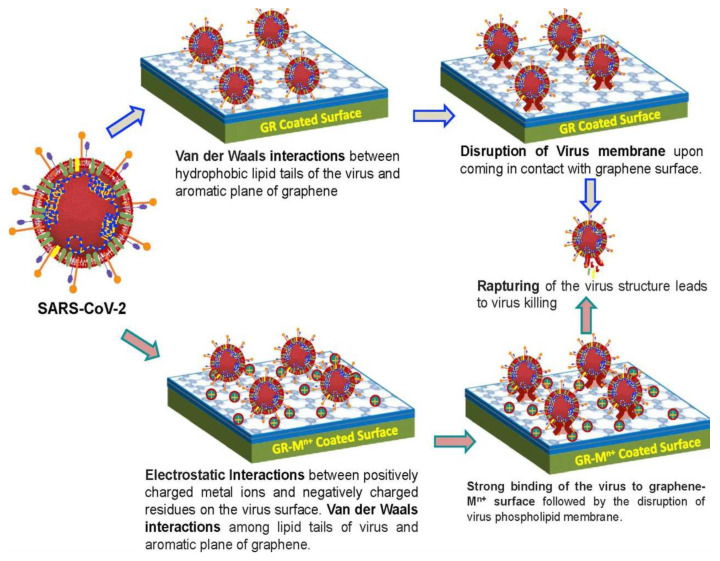
Representation of the probable role of G and G metal ion-coated surfaces in hindering substrate infection from viruses such as SARS-CoV-2. Taken from [120].

G and related materials can also play a key role in the global competition against viral illnesses such as COVID-19 by designing sensitive biosensors and diagnostic systems [119]. They can control virus spread and transmission via development of antiviral surfaces/coatings, nanofoams for facemasks, and 3D-printed components. Principally, for the SAR-CoV-2, since the virus structure is rich in carboxylic acid groups, GO/rGO-SO_3_ coatings on polymeric substrates such as polyvinylpyrrolidone (PVP) or poly(diallyl dimethylammonium chloride) (PDDA) could be developed [120]. Nanocomposites with antimicrobial metals such as Ag, Cu, Ti, and Au could also be examined for the manufacture of active antiviral coatings. These nanocomposites can aid in the capture and destruction of the virus structure and lessen its survival time on different substrates via diverse types of interactions, as schematized in Scheme 4.

Carbon nanomaterials have also been broadly investigated for water treatment, in particular for the manufacture of novel filtration membranes [121]. However, the complex procedure for the development of carbon-based adsorbents hinders their real uses. To resolve these issues, polymeric membranes incorporating carbon nanomaterials have been developed for different applications such as ultra- or nano-filtration, forward or reverse osmosis desalination, wastewater treatment, and so forth [122,123,124]. For instance, polyether sulfone (PES)/GO, PES/rGO, and polyethyleneimine (PEI)-wrapped GO membranes prepared through a simple mixing approach that can be applied for wastewater filtration [125]. PVDF membranes covalently functionalized with GO quantum dots also exhibited antibiofouling properties and outstanding antibacterial action versus *E. coli* [126], and they could be used for water treatment.

Nitrocellulose membrane filters wrapped with PVK:SWCNT (97:3 wt%) nanocomposites exhibited important antimicrobial action versus Gram-positive and Gram-negative bacteria (∼80–90%), and they showed a virus elimination effectiveness close to 2.5 logs [127]. A possible mechanism of cellular inactivation was bacteria cell membrane damage, since higher effluence of DNA was measured in the rest of the nanocomposite compared to the control. Their toxicity was assessed versus fibroblasts, and no toxic effects were found. These nanocomposites are more suitable than pure SWCNTs’ coated membranes for drinking water treatment, given that the lower amount of SWCNTs in the nanocomposite will decrease price and minimize toxicity.

## 5. Conclusions 

Carbon-based nanomaterials such as fullerenes, CNTs, and G and its derivatives GO and rGO, show outstanding antibacterial activity, and hence, they are suitable for manufacturing innovative nanocomposites for a wide range of applications from medicine to the food industry. These nanostructures utilize their antibacterial characteristics via physical damage (i.e., disruption of the cellular membrane) as well as via chemical impairment (phospholipid oxidation via ROS production). However, their practical applications as antimicrobial agents have not been completely explored yet, owing to their relatively poor dispersibility, expensiveness, and scalability challenges. To address these challenges, they can be incorporated within polymeric matrices, which also display antimicrobial activity in some cases.

This review recapitulates the state of the art in the research of polymeric nanocomposites with carbon nanomaterials as antimicrobial agents to fight against bacteria. Illustrative examples have been selected with the intent to clarify the potential modes of action and provide a better understanding of the antibacterial capabilities of polymeric nanocomposites with carbon-based nanostructures owing to synergistic effects. 

## 6. Future Perspectives 

To date, numerous inquiries regarding the antibacterial activity of carbon-based polymeric nanocomposites remain unanswered, including the specific mechanisms of action and the consequences of nanostructure size, loading, amount of surface groups, and so forth on the restriction of bacterial spread. Commonly, the antimicrobial properties have been assessed versus model pathogenic bacteria such as *E. coli* and *S. aureus*. Nevertheless, considering the increasing spread of antibiotic-resistant bacteria, it is critical to examine other microbes to demonstrate the wide variety of bactericidal properties of carbon nanomaterials. Besides, a more comprehensive and reliable understanding of the mechanisms of antimicrobial activity of this family of nanomaterials is critical. Another critical issue is that the procedures to synthesize carbon nanomaterials at an industrial level are very scarce and hence are highly desirable with a view towards using them in practical commercial applications. A simple and straightforward method to meet the strong demand for carbon nanomaterials via a sustainable approach with high yield is lacking. 

Another restraint on the real use of this type of nanocomposites is that to date, no in vivo test has been applied to animals. Though carbon nanomaterials may be suitable for several biomedical applications including wound healing, forthcoming uses in vivo cannot be predicted. One significant query that has to be addressed is if carbon nanomaterials can specifically target pathogenic microbes without disturbing mammal cells or non-pathogenic bacteria. Very few investigations regarding discriminatory killing have been reported so far. Additionally, the toxicity of carbon nanomaterials is still not well documented. Despite considerable struggles in evaluating the consequence of these nanomaterials on humans, results are frequently unreliable. This matter should be investigated in more detail, since the effects of carbon nanomaterials may be strongly influenced by their inherent properties. 

Overall, investigation in this arena is still in its childhood. The combination of these carbon nanomaterials and polymers offers novel perspectives owing to cooperation behavior. However, more research is required to assure that they are nontoxic. It can be foreseen that with exhaustive research and unceasing efforts, polymer nanocomposites with carbon nanomaterials will offer a novel perspective for the progress of antimicrobial agents. 

## Figures and Tables

**Table 1:** Comparison between the antimicrobial activity of nanoparticles, antibiotics, and polymer nanocomposites with carbon-based nanomaterials.

Matrix	Antibacterial Agent	Processing Method	Bacteria	Inhibition	Ref.
	(Shape or wt%)			(%)	
	SnO_2_ (rod-shaped)		*E. coli*	99.5	[11]
			*S. aureus*	99.5	
	Ag (spherical)		*E. coli*	90	[94]
–	Ag (rod-shaped)	–		100	[94]
	Ag (cuboctahedral and dodecahedral)		*E. coli*	100	
–		–	*P. Aeruginosa*	100	[95]
			*S. Aureus*	100	
	Cu (spherical)		*P. Aeruginosa*	99.5	[96]
–		–	*S. Aureus*	99.6	
			*B. subtilis*	99.6	
			*C. Albicans*	82.5	
			*P. Aeruginosa*	100	
–	CuO	–	*S. Aureus*	100	[97]
	(spherical)		*B. subtilis*	68	
			*C. Albicans*	65	
	ZnO (spherical)		*E. coli*	90	[89]
			*S. aureus*	85	
	TiO_2_ (spherical)		*E. coli*	98	[98]
			*S. aureus*	100	
	vancomycin		*S. aureus*	98.3 86	[99]
	TMP				
	penicillin		*S. aureus*	100	[100]
	tetracycline			100	
	chloramphenicol		*H.* *influenzae*	87.5	
	penicillin		*S. pneumoniae*	100	[101]
	oxacillin		*S. aureus*	88	
	amoxicillin		*H.* *influenzae*	100	[102]
			*Salmonella* spp.	100	
			*Shigella* spp.	100	
			*Corynebacteria spp*	100	
PMMA	GO-Ag (1) GO-Ag (2)	SN + curing	*E. coli*	–	[55]
			*S. aureus*		
			*S. mutans*		
PMMA fibers	GO (8)	pressurized gyration	*E. coli*	85	[57]
PNIPAM	Ag/G (0.5:1)	in situ polymerization	*E. coli* *S. aureus*	–	[58]
	Ag/G (1:1)				
	Ag/G (5:1)				
PNIPAM	GO/CNT (1:1)	free radical polymerization	*P. aeruginosa*	–	[59]
PVK	GO (3)	bulk polymerization	*B. subtilis*	89	[65]
			*R. opacus*	89	
			*E. coli*	89	
			*C. metallidurans*	91	
PVA	G (1)	SN+ solution casting	*E. coli*	92	[60]
	G (10)		*E.coli*	97.1	
	G (1)		*S. aureus*	92.3	
	G (10)		*S. aureus*	99.7	
PLA	GO/Ag (2)	in situ polymerization	*E. coli*	99	[69]
			*S. aureus*	99	
PVDF fibers	GO/Ag (1:0.5)	ES	*E. coli* *S. aureus*	–	[72]
	GO/Ag (1:1)				
	GO/Ag (1:2)				
PCL	GO/Ag (5:1)	SN+ solution casting	*E. coli*	59	[70]
PPF	PEG-GO (3)	SN+ curing	*S. aureus*	97	[12]
			*S. epidermidis*	94	
			*P. aeruginosa*	85	
			*E. coli*	81	
CS	GO/TiO_2_ (4/16)	SA	*A. niger*	99	[81]
			*B. subtilis*	99	
PHBV	CNC/GO (1:1)	solution casting	*E. coli*	99.7	[92]
			*S. aureus*	99.8	
AGAR	rGO/Ag/ZnO	solution casting	*S. aureus*	95	[77]
			*P. aeruginosa*	95	
AG	GO (1)	SN+ solution casting	*S. aureus*	99	[85]
			*S.epidermis*	99	
polyporphyrin	C60	electropolymerization	*S. aureus*	100	[46]
			*E. coli*	100	
PLL/PGA	SWCNT	LBL	*E.coli*	80	[47]
	SWCNT-PL-PEG		*E.coli*	90	
	SWCNT-PL-PEG		*S. epidermidis*	90	
PLGA	SWCNT (2)	solution casting	*E.coli*		[54]
			*S. epidermidis*	98	

SN: sonication; ES: electrospinning; SA: self-assembly; LBL: layer-by-layer TMP: trimethoprim-sulfamethoxazole.
